# Optimizing Bone Regeneration with Demineralized Dentin-Derived Graft Material: Impact of Demineralization Duration in a Rabbit Calvaria Model

**DOI:** 10.3390/jfb15110331

**Published:** 2024-11-06

**Authors:** Bounghoon Lee, Hyunsuk Choi, Dong-Seok Sohn

**Affiliations:** 1 Department of Dentistry and Oral and Maxillofacial Surgery, Daegu Catholic University Medical Center, Daegu 42472, Republic of Korea; bounghoon.lee.dds@gmail.com; 2 Department of Dentistry and Prosthodontics, Daegu Catholic University School of Medicine, Daegu 42472, Republic of Korea; hschoi@cu.ac.kr

**Keywords:** bone graft, bone regeneration, rabbit calvaria, histology, dentin-derived matrix

## Abstract

This study evaluated the regenerative potential of demineralized dentin-derived matrix (DDM) as a bone graft material in rabbit calvaria. DDM, sourced from extracted teeth, is emerging as an alternative to traditional grafts like allografts and xenografts. We aimed to identify the most effective demineralization protocol to optimize the regenerative capacity of DDM. Four groups were compared: a control group without grafts, a non-demineralized DDM group, and two demineralized DDM groups (15 and 30 min demineralization). Histomorphometric analysis was conducted in a randomized and blinded setting at 2, 4, and 8 weeks post-graft placement. The results revealed that the 15 min demineralized DDM group showed the most significant new bone formation (42.51% ± 6.40% at 8 weeks; *p* < 0.05), suggesting its potential as a highly effective regenerative graft material.

## 1. Introduction

Bone grafting is defined as a surgical procedure to replace missing bone with an artificial, synthetic, or natural substitute [1]. Tooth extraction, reported to be the most widely performed dental procedure, leads to substantial changes in alveolar ridge dimensions among patients [2]. These changes present major obstacles to achieving ideal reconstructive outcomes in modern implant dentistry. To address these challenges, horizontal or vertical bone augmentation is performed, aiming for multidimensional regeneration using various graft materials and surgical techniques [3].

Regenerative bone grafting materials are typically classified into four main types: autogenous, allogenic, xenogenic, and alloplastic. Autogenous bone grafts are considered the gold standard due to their ability to fulfill the essential components of the regenerative triad: osteoconduction, osteoinduction, and osteogenesis [4]. They also offer the advantage of reduced immunological reactions [5]. However, autogenous bone grafts have drawbacks, including potential complications at the donor site, unpredictable bone resorption, and limited availability [6,7,8,9].

In contrast, allogenic and xenogenic grafts do not require additional surgery for preparation or harvesting, but they may exhibit batch variability and carry risks of disease transmission and immunologic rejection [9,10]. Synthetic materials provide precise control over characteristics such as porosity and hydrophilicity or hydrophobicity through controlled manufacturing processes. However, their efficacy often depends on the addition of bioactive factors, such as platelet-rich plasma, to facilitate regeneration [10].

To address the limitations of autogenous grafts and find viable alternatives with comparable biological properties, researchers have explored the use of extracted human teeth as novel graft materials. The osteogenic capacity of demineralized teeth was first recognized in 1967 [11,12]. Given their potential to fulfill the regeneration triad and overcome the limitations associated with autogenous bone grafts, tooth-derived bone graft material has been considered and utilized as a promising regenerative bone graft material for decades [13].

Teeth possess physicochemical properties similar to bone, particularly dentin, which has a comparable chemical composition. Dentin is composed of 70–75% inorganic material, 20% organic material, and 10% water, while alveolar bone comprises 65% inorganic material, 25% organic material, and 10% water [13,14,15,16,17,18,19,20,21,22]. Both human bone and dentin also contain key growth factors such as bone morphogenetic proteins (BMP), transforming growth factor-beta (TGF-β), and insulin-like growth factor II (IGF-II), which are essential for bone induction through endochondral bone differentiation [23,24].

The use of demineralized teeth as bone grafting material has a rich history, dating back to 1967. Demineralized teeth have long been acknowledged for their high osteoinductive and osteoconductive properties [12,13,21,25,26]. Importantly, both autogenous and allogenic sources of demineralized tooth-derived bone grafts exhibit minimal immune response and antigenicity [11,12,13,27,28]. Over decades of research, demineralized dentin-derived matrix has proven to be an effective graft material, often outperforming other popular grafts like Bio-Oss, a bovine bone primarily consisting of hydroxyapatite, and B-TCP, a synthetic bone graft material made from calcium phosphate ceramic, in regenerative efficacy [29,30].

The demineralization process significantly enhances the regenerative properties of dentin-derived matrix by reducing antigenicity and immunogenicity while accelerating bone formation [28,31,32]. It upregulates bone regeneration by serving as a carrier for BMP-2 and providing crucial cell adhesion sequences [14]. Various studies have demonstrated that non-demineralized dentin-derived matrix can lead to delayed osteoinduction due to hydroxyapatite obstructing the release of growth factors [33]. In contrast, demineralization facilitates the release of these factors, thereby promoting faster and more effective bone regeneration [31]. Additionally, demineralized dentin-derived matrix, particularly when used in block forms or with perforations, shows more rapid new bone formation compared to other graft materials [32]. However, excessive demineralization is known to increase graft resorption, possibly due to the enzymatic breakdown of the exposed collagen matrix [34].

The demineralization durations in this study—specifically, 15 and 30 min—were selected based on practical clinical considerations rather than prior empirical research. These timeframes align with efficient and feasible protocols in clinical settings, where time-sensitive preparation methods are beneficial for patient care. By focusing on clinically relevant demineralization times, this study aims to bridge laboratory research with practical application in dental and maxillofacial practices, providing insights that can be readily translated into patient treatment.

To determine the optimal degree of demineralization for maximizing the bone regeneration potential of dentin-derived matrix, we conducted a histomorphometric analysis to evaluate new bone formation in rabbit calvaria using different demineralization protocols. These findings contribute to refining demineralization protocols for dentin-derived graft materials, which are already commercially available and widely used.

## 2. Materials and Methods

This animal study included 15 adult male New Zealand white rabbits, each weighing between 2.8 and 3.2 kg (average weight 3.0 kg). The study was approved by the Animal Care and Use Committee at the Catholic University Medical Center of Daegu (Approval No. DGIACUC-150911-14) and was conducted at the University Medical Center of Daegu from July 2016 to April 2017.

The rabbits were randomly assigned to three groups, with five rabbits in each group. All surgical procedures were identical for all animals. General anesthesia was administered intramuscularly using a combination of 30 mg/kg ketamine (Ketalar; Yuhan Co., Seoul, Republic of Korea) and 10 mg/kg xylazine (Rompun; Bayer Korea, Seoul, Republic of Korea). Additionally, a 0.5 mL subcutaneous injection of lidocaine with 1:100,000 epinephrine was given along the calvarial midline.

### 2.1. Surgical Procedures

The extracted human teeth were cleaned and sterilized prior to preparation. Enamel tissue was removed by sectioning the coronal portion of the teeth, and any old restorations, caries, calculus, and attached soft tissue debris were meticulously cleaned using manual and rotary instruments with extensive irrigation. The teeth were then crushed into smaller particles, approximately 0.8–1.0 mm in size, using a surgical mallet. Demineralization was performed using 0.6 N hydrochloric acid for either 15 or 30 min under vacuum compression with ultrasonic vibration in a vacuum-ultrasonic device (Vacua-Sonic System, CosmoBioMedicare Co., Seoul, Republic of Korea). The demineralized dentin-derived matrix particles were washed with phosphate-buffered saline (PBS), sterilized with a sterilization reagent, and rinsed with PBS and distilled water.

**Figure 1:**
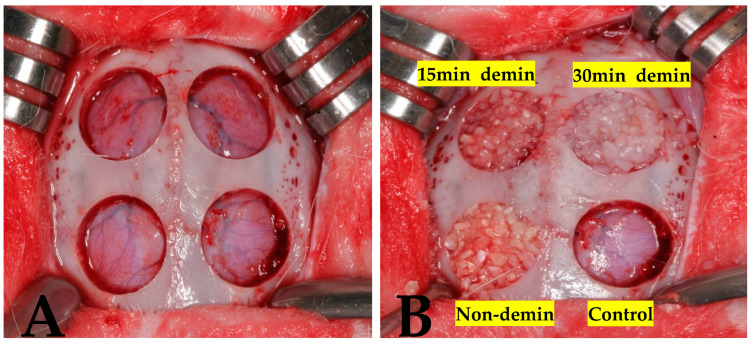
(**A**) Sites prepared before graft material placement. (**B**) Experimental group assignments.

The animals were stabilized on a surgical table, and periosteal incisions were made along the sagittal midline of the calvaria to expose the frontal bone. Four circular bone defects, each 8 mm in diameter, were prepared in each subject using trephine burs (Figure 1A). The resected bone segments were carefully removed to avoid injury to the underlying brain tissue. The defects were then assigned to four experimental groups: Group 1 (control group) was filled with blood coagulum; Group 2 (non-demin) was filled with non-demineralized dentin-derived matrix using a Dentin Grinder™ (KometaBio, Fort Lee, NJ, USA); Group 3 (15 min demin) was filled with 15 min demineralized dentin-derived matrix (Vacuasonic (CosmoBiomedicare, Seoul, Republic of Korea)); and Group 4 (30 min demin) was filled with 30 min demineralized dentin-derived matrix (Vacuasonic) (Figure 1B).

For wound closure, 4-0 nylon sutures (Blue nylon, Ailee Co., Busan, Republic of Korea) were utilized. All subjects received intramuscular antibiotics (20 mg/kg gentamicin [Donghwa Co., Seoul, Republic of Korea]) for 3 days following the surgical procedure. The rabbits in each group were euthanized at 2 weeks (*n* = 5), 4 weeks (*n* = 5), or 8 weeks (*n* = 5) post-surgery.

### 2.2. Tissue Preparation

The rabbits were euthanized at 2, 4, and 8 weeks under general anesthesia. The calvaria were carefully excised from the cranium using a microsaw and fixed in neutral-buffered formalin for 24 h. The specimens were then washed with 0.1 M phosphate buffer, demineralized in 10% formic acid for 10 days, embedded in paraffin (Paraplast; Oxford, MS, USA), and sectioned into 5 μm thick serial slices through the center of the circular preparation. The sections were stained with hematoxylin-eosin and Masson’s trichrome staining. Changes in newly formed bone and soft tissue in the calvaria preparations were examined under a light microscope.

### 2.3. Histomorphometric Analysis

Ten randomly selected specimens from each group were photographed using an AxioCam MRc5 and Axiophot Photomicroscope (Carl Zeiss, Oberkochen, Germany). The images were analyzed using AxioVision SE64 software (Carl Zeiss, Oberkochen, Germany).

The following histomorphometric measurements were assessed: the total area of augmentation, the area of new bone formation, the turnover rate of regenerative graft materials, and the bone marrow space dimensions. The total area of augmentation included the area of new bone formation, graft material, fibrous tissue, and vascular tissue within the preparation. The percentage of new bone formation and the types of regenerative graft materials used were analyzed to evaluate the efficacy of new bone formation based on different degrees of demineralization.

### 2.4. Statistical Analysis

Data processing and statistical evaluation were conducted using SPSS software (version 25.0, SPSS Inc., Chicago, IL, USA). Differences within and between groups were assessed using one-way analysis of variance (ANOVA) with Tukey’s post hoc test to evaluate statistical significance among multiple groups. Results are presented as means ± standard deviation, with a *p*-value of less than 0.05 considered statistically significant. Control group data were included in analyses where appropriate; however, control values were not applicable or available in all figures. Thus, certain figures emphasize treatment groups for statistical comparisons across demineralization protocols.

## 3. Results

### 3.1. Histological Analysis

Masson’s trichrome staining revealed host or lamellar bone in red, while woven or newly formed bone appeared blue. Regenerative graft material particles exhibited various colors, which allowed for clear differentiation from the surrounding tissue. Notably, no signs of inflammation were observed in any experimental group under light microscopy.

### 3.2. Two-Week Results

**Figure 2:**
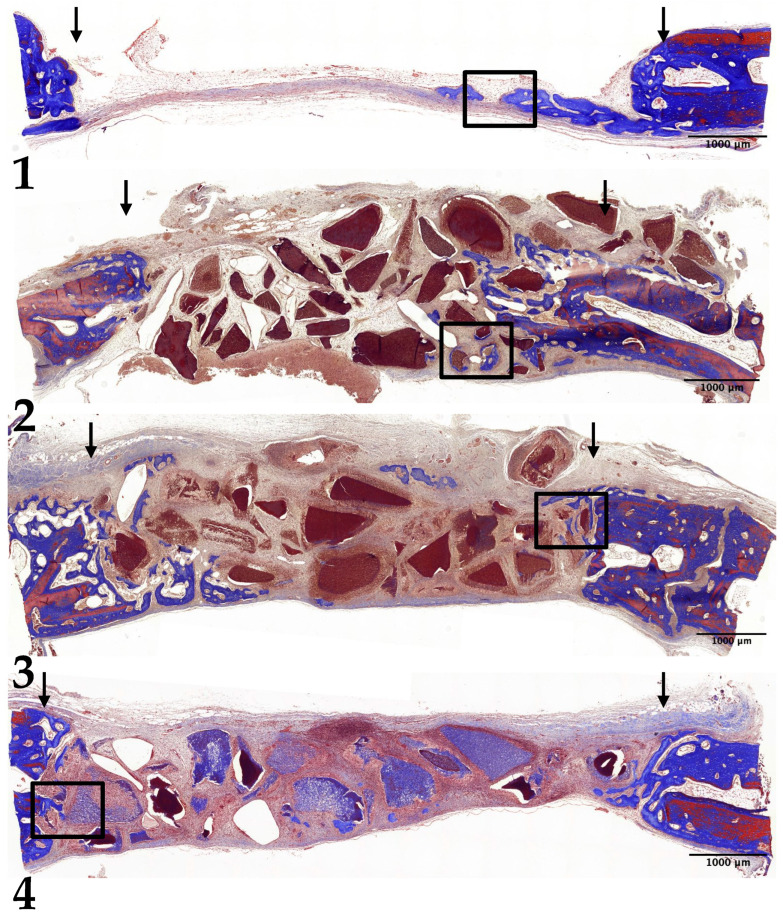
Low-magnification images of rabbit calvaria at 2 weeks post-surgery: Group (1) (control), Group (2) (non-demin), Group (3) (15 min demin), and Group (4) (30 min demin). Arrows indicate preparation margins. Scale bar = 1000 µm. Boxed areas magnified in Figure 3. (Masson’s trichrome stain, ×20).

**Figure 3:**
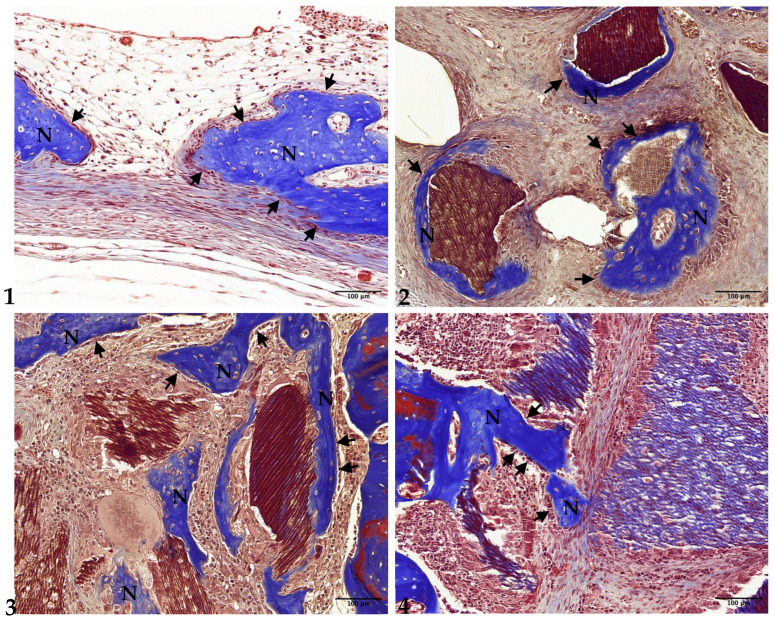
Photomicrograph depicting new bone formation at 2 weeks post-surgery: Group (1) (control), Group (2) (non-demin), Group (3) (15 min demin), and Group (4) (30 min demin). Scale bar = 100 µm. N denotes newly formed bone. Arrows indicate osteoblasts. (Masson’s trichrome stain, ×200).

At the 2-week mark, all experimental groups showed limited new bone formation, primarily concentrated around the margins of the preparations. The amount of newly formed bone was consistent across all groups. In Group 1, the center of the preparation appeared somewhat depressed and flattened, influenced by the surrounding connective tissue and dura mater. Newly formed bone was partially visible along the surface of the preparation margin, with osteoblasts identified on the surface of this new bone (Figure 2(1) and Figure 3(1)).

Groups 2, 3, and 4 exhibited similar findings, with partial new bone formation observed at the preparation margins and on the surfaces of the graft particles: Group 2 (Figure 2(2) and Figure 3(2)), Group 3 (Figure 2(3) and Figure 3(3)), and Group 4 (Figure 2(4) and Figure 3(4)). Osteoblasts were present on the surface of the newly formed bone in all groups.

### 3.3. Four-Week Results

**Figure 4:**
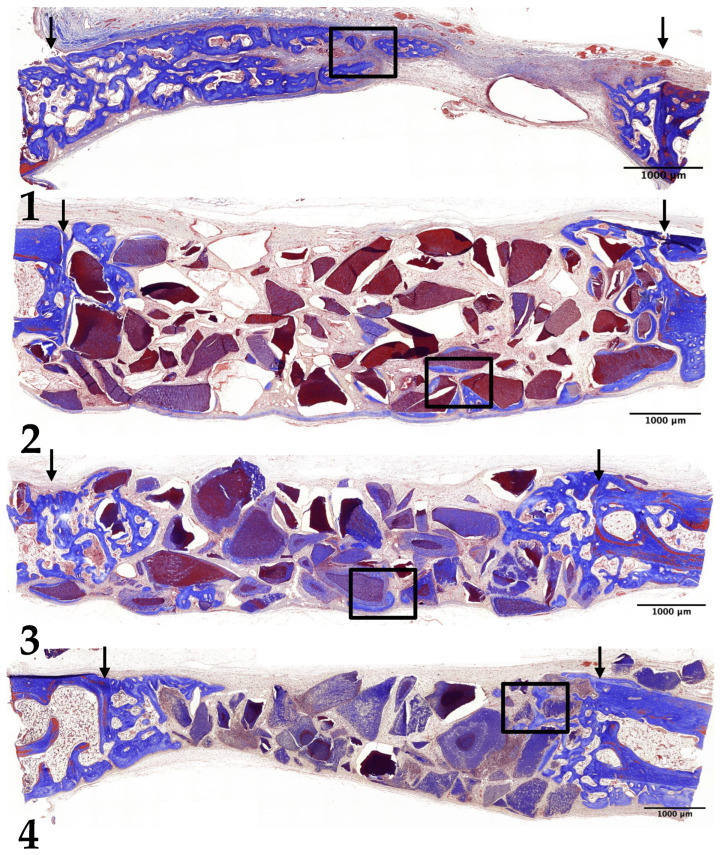
Low-magnification images of rabbit calvaria at 4 weeks post-surgery: Group (1) (control), Group (2) (non-demin), Group (3) (15 min demin), and Group (4) (30 min demin). Arrows indicate preparation margins. Scale bar = 1000 µm. Boxed areas magnified in Figure 5. (Masson’s trichrome stain, ×20).

**Figure 5:**
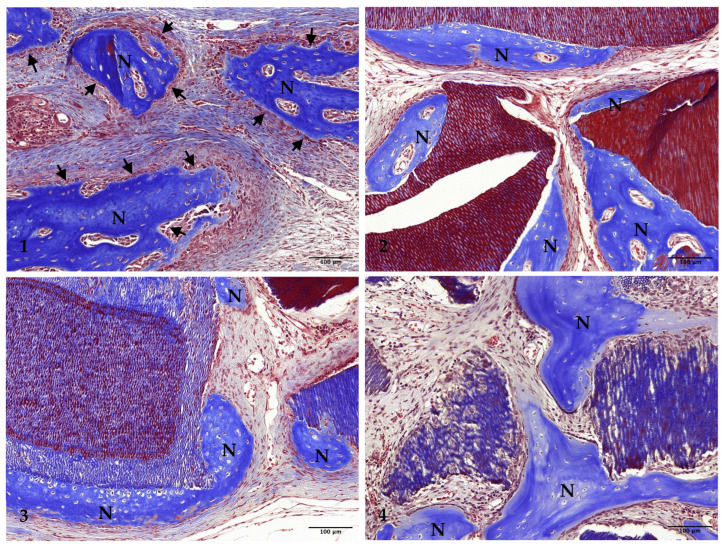
Photomicrograph showing new bone formation at 4 weeks post-surgery: Group (1) (control), Group (2) (non-demin), Group (3) (15 min demin), and Group (4) (30 min demin). Scale bar = 100 µm. N represents newly formed bone. Arrows indicate osteoblasts. (Masson’s trichrome stain, ×200).

In Group 1, the newly formed bone had thickened, extending to the center of the preparation. A significant number of osteoblasts were observed on the surface of the newly formed bone (Figure 4(1) and Figure 5(1)).

In Group 2, the thickness of the newly formed bone had increased, particularly on the surfaces of the undemineralized dentin-derived graft particles. The density of the newly formed bone was higher compared to the 2-week results. However, most of the new bone formation remained concentrated near the preparation margins (Figure 4(2) and Figure 5(2)).

In Group 3, both the thickness and density of the newly formed bone had increased compared to the 2-week period. Notably, new bone formation was now also observed at the center of the defect. The size and density of the 15 min demineralized dentin-derived graft particles remained consistent with the 2-week results (Figure 4(3) and Figure 5(3)).

In Group 4, the newly formed bone had also thickened and was observed on the surfaces of the 30 min demineralized particles. The density of the newly formed bone had increased compared to the 2-week results. However, similar to Group 2, the majority of new bone formation was still localized near the preparation margins. The density of the 30 min demineralized graft particles remained similar to the 2-week results (Figure 4(4) and Figure 5(4)).

### 3.4. Eight-Week Results

**Figure 6:**
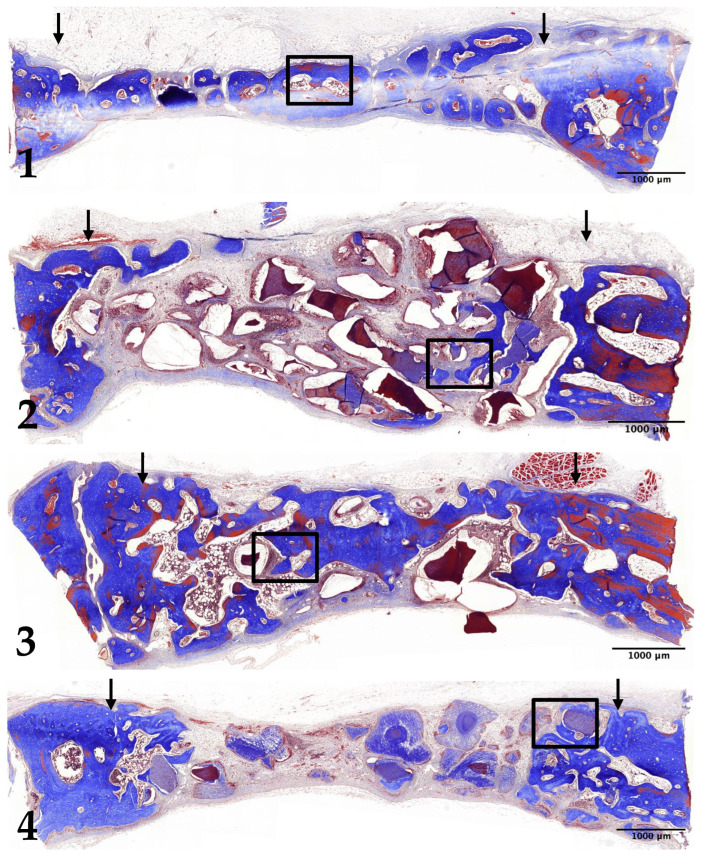
Low-magnification images of rabbit calvaria at 8 weeks post-surgery: Group (1) (control), Group (2) (non-demin), Group (3) (15 min demin), and Group (4) (30 min demin). Arrows indicate preparation margins. Scale bar = 1000 µm. Boxed areas magnified in Figure 7. (Masson’s trichrome stain, ×20).

**Figure 7:**
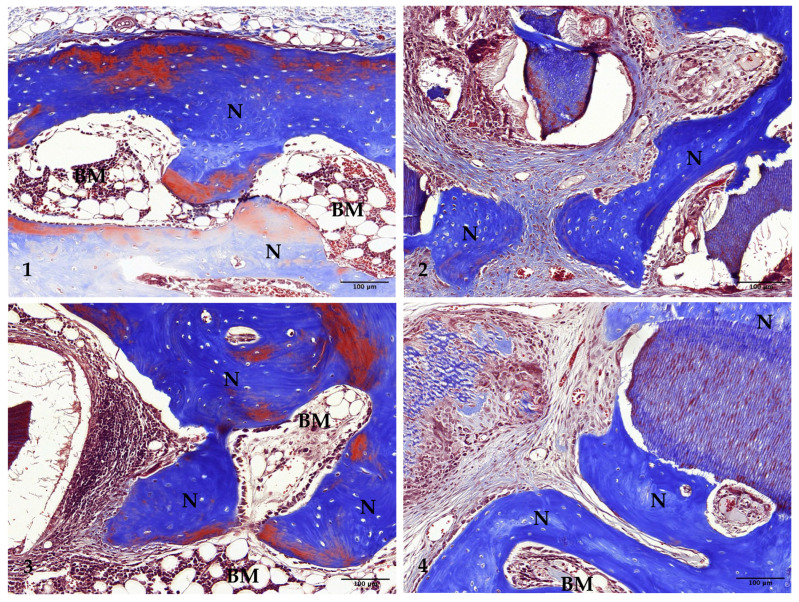
Photomicrograph illustrating new bone formation at 8 weeks post-surgery: Group (1) (control), Group (2) (non-demin), Group (3) (15 min demin), and Group (4) (30 min demin). Scale bar = 100 µm. N denotes newly formed bone; BM denotes bone marrow. (Masson’s trichrome stain, ×200).

In Group 1, new bone formation extended from the margins to the center of the preparation. The newly formed bone was notably thicker, and bone marrow spaces containing adipose tissue were observed interspersed among the new bone cells (Figure 6(1) and Figure 7(1)).

In Group 2, thicker bone formation was evident, particularly on the surfaces of the undemineralized dentin-derived graft particles. The density of the newly formed bone had increased compared to the 4-week results. However, new bone formation was absent at the center of the preparation, suggesting limited central integration (Figure 6(2) and Figure 7(2)).

In Group 3, there was a substantial increase in both the thickness and density of the newly formed bone compared to the 4-week results. Bone growth extended from the margins to the center of the preparation, indicating more uniform regeneration. Some mature lamellar bone cells were observed within the new bone, and an increased number of bone marrow spaces containing adipose tissue were present between the new bone cells. However, the size and density of the 15 min demineralized dentin-derived graft particles had decreased, suggesting the ongoing resorption and integration of the graft material (Figure 6(3) and Figure 7(3)).

In Group 4, new bone formation on the surfaces of the 30 min demineralized graft particles had increased compared to the 4-week results. Despite this, there was no significant increase in bone formation at the center of the preparation, indicating that central regeneration remained limited. The density of the 30 min demineralized graft particles had decreased, similar to the 15 min group, and bone marrow spaces containing adipose tissue were present between the newly formed bone cells and the graft particles, reflecting ongoing remodeling processes (Figure 6(4) and Figure 7(4)).

### 3.5. Histomorphometric Analysis: New Bone Formation

In Group 2, the ratios of newly formed bone area to augmented area were 4.42% ± 1.06% at 2 weeks, 8.58% ± 1.11% at 4 weeks, and 10.70% ± 2.58% at 8 weeks. For Group 3, these ratios were 6.59% ± 1.76% at 2 weeks, 15.80% ± 3.28% at 4 weeks, and 42.51% ± 6.40% at 8 weeks. In Group 4, the corresponding ratios were 5.61% ± 1.48%, 11.81% ± 2.09%, and 21.80% ± 1.18% at the same time points.

**Figure 8:**
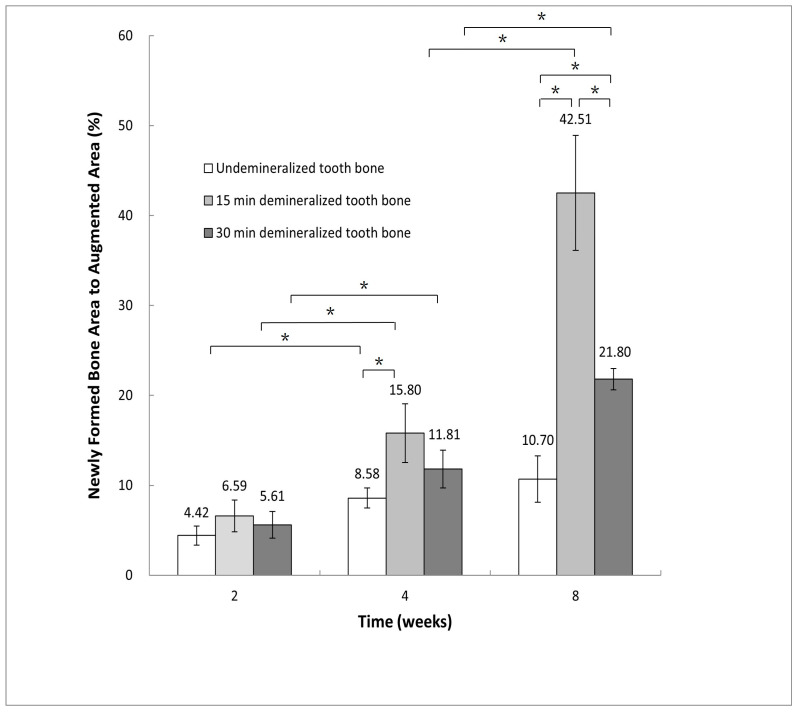
Histomorphometric measurement of the newly formed bone area to augmented area ratio at 2, 4, and 8 weeks (control data not available for these comparisons) (* *p* < 0.05).

Statistical analysis using one-way ANOVA followed by post hoc comparisons revealed a statistically significant increase in the new bone area at 8 weeks compared to 2 and 4 weeks for both Groups 3 and 4 (*p* < 0.05). However, in Group 2, the increase in new bone area at 8 weeks was not statistically significant when compared to the earlier time points (*p* < 0.05) (Figure 8).

At the 4-week mark, Group 3 exhibited significantly greater new bone formation compared to Group 2. By 8 weeks, Group 3 showed the highest amount of new bone formation among all experimental groups, followed by Groups 4 and 2 (Figure 8).

### 3.6. Histomorphometric Analysis: Decrease in Remaining Graft Material Area

The ratios of graft material area to total augmented area in Group 2 were 34.34% ± 4.96% at 2 weeks, 31.33% ± 4.02% at 4 weeks, and 25.20% ± 4.35% at 8 weeks. In Group 3, these ratios were 35.42% ± 4.46% at 2 weeks, 26.82% ± 4.05% at 4 weeks, and 12.31% ± 4.53% at 8 weeks. For Group 4, the corresponding ratios were 33.12% ± 4.29%, 30.94% ± 6.16%, and 21.60% ± 4.58% at the same time points.

**Figure 9:**
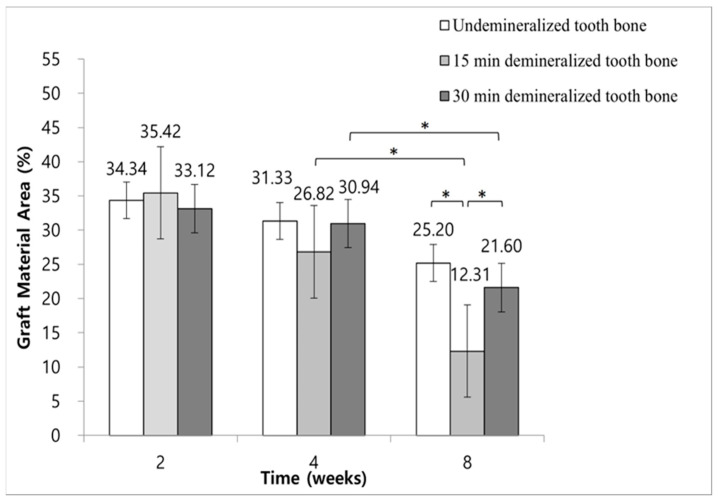
Histomorphometric measurement of the graft material to total augmented area ratio at 2, 4, and 8 weeks (* *p* < 0.05).

A statistically significant decrease in the ratio of graft material area to total augmented area was observed in Groups 3 and 4 at 8 weeks, compared to the ratios at 2 and 4 weeks (*p* < 0.05). Conversely, Group 2 did not show a significant difference in this ratio at the 8-week mark compared to the earlier time points (*p* < 0.05) (Figure 9).

At 8 weeks, Group 3 exhibited a significantly greater reduction in the ratio of graft material to total augmented area compared to Groups 2 and 4. Additionally, Group 4 had a significantly lower ratio of graft material to total augmented area at 8 weeks compared to Group 2 (Figure 9).

### 3.7. Histomorphometric Analysis: Quantifying Changes in Bone Marrow Area

**Figure 10:**
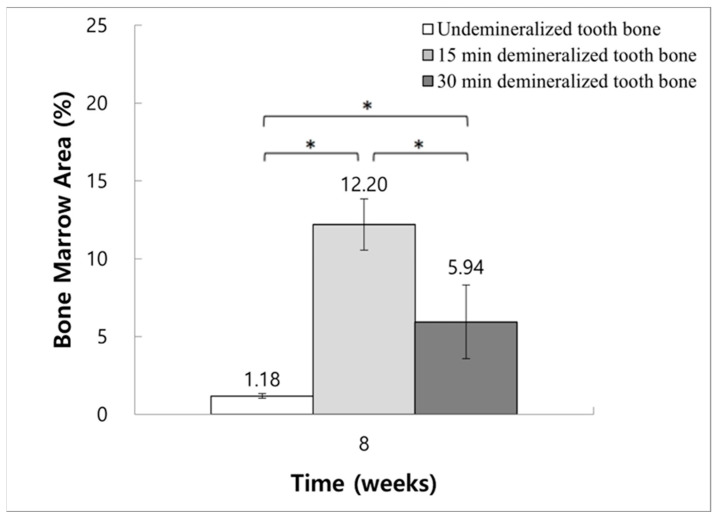
Histomorphometric measurement of the bone marrow area to total augmentation area ratio at 8 weeks (control data not available for these comparisons) (* *p* < 0.05).

At the 8-week evaluation point, the ratios of bone marrow area to the total augmented area were as follows: Group 2 reported 1.18% ± 0.16%, Group 3 demonstrated a significantly higher ratio of 12.20% ± 1.64%, and Group 4 had 5.94% ± 2.37%. The increase in bone marrow area within Group 3 was notably significant when compared to Groups 2 and 4, highlighting the differential effects of treatment modalities across the groups (Figure 10).

## 4. Discussion

The importance of selecting an appropriate regenerative graft material and optimizing its preparation for clinical application cannot be overstated in achieving successful regeneration outcomes. In this study, we assessed the effectiveness of new bone formation in rabbit calvaria using various graft materials: absence of graft material (Group 1), non-demineralized dentin-derived graft material (Group 2), 15 min demineralized dentin-derived graft material (Group 3), and 30 min demineralized dentin-derived graft material (Group 4).

Identifying an optimal preparation protocol for tooth-derived graft materials is essential to achieving consistent clinical results. Here, the ratio of newly formed bone to the total augmented area served as a measure of each graft material’s regenerative capacity, with higher ratios indicating faster bone formation and greater regeneration efficiency. Histomorphometric analysis revealed new bone formation in all groups, including the control group without graft material. However, statistically significant new bone formation was observed only in the demineralized graft groups (Groups 3 and 4) compared to the non-demineralized (Group 2) and no-graft groups (Group 1). Notably, the 15 min demineralized group (Group 3) exhibited significantly more new bone formation than all other groups, including Group 4, at 8 weeks. These findings support the advantages of demineralization, with the 15 min protocol yielding superior results compared to the 30 min protocol, aligning with prior studies demonstrating more effective bone formation from demineralized dentin than from undemineralized dentin [31,33].

The ratio of graft material area to the total augmented area was also analyzed to assess graft material turnover, where lower ratios indicate a higher turnover rate and, therefore, more efficient remodeling. Our analysis showed a clear advantage of demineralization: both the 15 min and 30 min demineralization groups exhibited faster graft turnover compared to the non-demineralized group, with the 15 min group outperforming the 30 min group at 8 weeks. Thus, demineralization facilitated faster graft material turnover, with the 15 min protocol proving optimal for enhancing both new bone formation and graft remodeling.

As demonstrated in a recent study, dentin exhibits superior osteoblastic activity and cellular proliferation compared to other tooth structures, such as enamel and periodontal ligaments [35]. The demineralization process of dentin-derived matrix further enhances these regenerative properties by reducing antigenicity and immunogenicity while promoting bone formation [28,31,32]. This process removes crystalline inorganic substances from dentinal tubules, facilitating resorption and enabling effective bone remodeling [36]. Hydrochloric acid treatment plays a key role in releasing growth factors like BMP and TGF-β; however, balancing effective demineralization with protein stability is essential. Excessive demineralization, as observed in the 30 min group, may result in the partial degradation of osteoinductive proteins, potentially reducing their regenerative efficacy. The results of this study suggest that selecting an optimal demineralization duration can maximize protein bioavailability while preserving osteoinductive potential, highlighting the importance of a carefully calibrated demineralization protocol [37].

Furthermore, a higher ratio of bone marrow area to the total augmented area reflects the efficacy of the regenerative graft material and indicates regeneration progression. Histomorphometric analysis revealed a significant advantage in the 15 min demineralization group (Group 3) over both the 30 min demineralization and non-demineralized groups (Groups 4 and 2, respectively), underscoring the importance of identifying the optimal demineralization level for preparing dentin-derived grafts.

In Group 1, autogenous bone regeneration occurred, though with less volume than in other groups, suggesting that natural wound healing can regenerate some bone volume without additional graft material. Additionally, in Group 4, noticeable graft volume loss was observed due to excessive demineralization. Nevertheless, Group 4 yielded more bone regeneration than the control, reinforcing that dentin-derived matrix as a graft material enhances regeneration outcomes even without optimal demineralization.

This study has several limitations. First, although the rabbit calvaria model is widely used due to its reproducibility and established protocols, it may not fully replicate the critical bone defects encountered clinically. Spontaneous bone formation observed in some controls underscores this limitation, suggesting that alternative models with larger or more anatomically relevant sites could better reflect clinical outcomes in future studies.

Second, while using human teeth as a bone graft material offers advantages as an alternative to allografts and xenografts, challenges remain in mass production and processing. Using extracted teeth from the same individual as the graft recipient is straightforward, but replacing allogenic bone grafts would require overcoming ethical and cultural considerations related to tooth donation. Further research on public perception and acceptability would support broader application.

Third, although immunohistochemical analysis could offer deeper insights into protein expression and cellular responses within the graft material, it was not included in this study. Future research could employ immunohistochemistry to evaluate specific growth factors or cell markers, such as BMP and TGF-β, to provide further clarity on DDM’s osteoinductive mechanisms. Additionally, advanced analytical techniques—such as morphometric analysis of angiogenesis, dual-energy X-ray absorptiometry (DEXA), and spectroscopy methods like FTIR and Raman—could yield more comprehensive insights into bone formation processes.

Lastly, incorporating bioactive additives, such as platelet-rich fibrin or concentrated growth factors, into dentin-derived graft materials could enhance healing. Future studies should investigate the efficacy of these bioactive additives in conjunction with differently prepared dentin-derived matrix grafts to optimize clinical outcomes.

## 5. Conclusions

The findings of this study, along with the extensive and proven track record of tooth-derived graft materials, suggest that it is time to reconsider the current gold standard in regenerative bone grafting. Extracted tooth-derived regenerative bone grafting materials, particularly when prepared with optimized demineralization processes, are already well-established, commercially available, and utilized daily in clinical settings worldwide. This material, far from being experimental, has demonstrated consistent and predictable outcomes, offering significant advantages in cost-effectiveness, enhanced efficacy through the recycling of biological waste, and greater patient comfort and acceptance due to its autogenous origin. These benefits make it a compelling alternative to allografts and xenografts, which can raise cultural or religious concerns. Given its widespread use and ongoing research, the demineralized dentin-derived matrix represents not just an emerging standard but a well-validated option with exciting potential for the future of regenerative grafting.

## Data Availability

The original data presented in the study are openly available in FigShare at doi:10.6084/m9.figshare.27214965.
